# A comprehensive multidisciplinary approach for identifying asbestos exposure among underground workers

**DOI:** 10.47626/1679-4435-2024-1271

**Published:** 2025-01-31

**Authors:** Alejandro Salvado, Lilian Capone, Paula Zamorano, Mayra Samudio, María Teresa Garcia-de-Dávila, Glenda Ernst

**Affiliations:** 1 Pneumonology, Hospital Británico, Buenos Aires, Argentina; 2 Respiratory Occupational Pathology, Faculty of Medicine, University of Buenos Aires, Buenos Aires, Argentina; 3 Image, Hospital Británico, Buenos Aires, Argentina; 4 Anatomical Pathology, Hospital Británico, Buenos Aires, Argentina; 5 Independent Researcher, Consejo Nacional de Investigaciones Científicas y Técnicas, Buenos Aires, Argentina

**Keywords:** asbestos, occupational exposure, prevalence, risk factor, amianto, exposição ocupacional, prevalência, fatores de risco

## Abstract

**Introduction:**

Inhalation of asbestos fibers can lead to a range of diseases, including asbestosis, pleural plaques, lung cancer, and malignant mesothelioma. Despite regulatory efforts, asbestos-related diseases remain a significant public health issue.

**Objectives:**

This study aimed to assess the characteristics and prevalence of asbestos-related diseases among exposed workers.

**Methods:**

We conducted a descriptive cohort study with underground workers in Buenos Aires, Argentina, from March 2018 to March 2023. A comprehensive screening and surveillance program, including medical examinations, was implemented to identify exposure-related signs and symptoms. Histological sections from paraffin-embedded tissue blocks were analyzed using light and polarization microscopy for lung cancer cases.

**Results:**

A total of 2,690 participants were included, of whom 2.8% (n = 77) had asbestos-related diseases and 0.22% (n = 6) had lung cancer. Occupational exposure exceeding 20 years was significantly associated with an elevated risk of asbestos-related diseases (odd ratio: 3.02; 95%CI 1.7-5.3).

**Conclusions:**

The prevalence of occupational diseases among underground workers was consistent with findings from other surveillance programs for asbestos-exposed workers. Occupational exposure exceeding 20 years emerged as a significant risk factor, markedly increasing the likelihood of asbestos-related diseases

## Abstract (pt)

**Introdução:**

A inalação de fibras de amianto pode causar inúmeras doenças, incluindo asbestose, placas pleurais, câncer de pulmão e mesotelioma maligno. Apesar das iniciativas regulatórias, as doenças associadas ao amianto ainda são um problema significativo de saúde pública.

**Objetivos:**

Este estudo pretende avaliar as características e a prevalência de doenças causadas pelo amianto em trabalhadores expostos.

**Métodos:**

Trata-se de um estudo de coorte descritivo sobre trabalhadores do metrô de Buenos Aires, de março de 2018 a março de 2023. Por meio de um amplo programa de triagem e vigilância, foram realizados exames médicos para identificar sinais e sintomas relativos à exposição. Em quatro casos de câncer de pulmão, usamos blocos de tecido embebidos em parafina e os examinamos em cortes histológicos com microscopia de luz e polarização.

**Resultados:**

O estudo incluiu 2.690 indivíduos, revelando que 2,8% (n = 77) dos trabalhadores tinham doenças relacionadas ao amianto e 0,22% (n = 6) desenvolveram câncer de pulmão. Observou-se um risco significativamente alto associado à exposição ocupacional superior a 20 anos (razão de chances: 3,02; IC95% 1,7 a 5,3).

**Conclusões:**

A prevalência de doenças ocupacionais em trabalhadores de subsolo foi compatível com os resultados de outros programas de vigilância de trabalhadores expostos ao amianto. A exposição ocupacional superior a 20 anos foi considerada um fator de risco significativo, aumentando sobremaneira a probabilidade de doenças associadas ao amianto.

## INTRODUCTION

Asbestos refers to a group of mineral silicates, frequently used in construction due to their structural strength and heat-resistant properties. There are two types of asbestos fibers, namely serpentine and amphibole, each with distinct physical and chemical characteristics.^1,2^ Repeated inhalation of these fibers increases the risk of pleuropulmonary diseases, asbestosis, and lung cancer. Most of these conditions have a prolonged latency period.^3,4^

Pleuropulmonary diseases can manifest as either malignant or non-malignant. Malignant mesothelioma, a rare tumor in the general population, is associated with long-term exposure to asbestos, especially amphibole fibers.^5^ More than 85% of cases of mesothelioma are associated with occupational exposure.^3^ The non-malignant forms of asbestos may present as pleural plaques, diffuse pleural thickening, benign pleural effusion, and rounded atelectasis. Benign pleural disease typically has a long latency period, usually more than 20 years.^6^ In populations environmentally exposed to asbestos, the prevalence of benign pleural disease ranges from 0.53% to 8%, increasing proportionally with the years of exposure.^7^

Asbestosis is characterized by diffuse interstitial fibrosis secondary to inhalation of asbestos fibers. Symptoms generally appear after a latency period of 20-30 years and include digital clubbing, Velcro crackles, and dry cough. The most common comorbidity in patients with asbestosis is pleural plaques (96%).^8^

Asbestos exposure has also been reported to increase the risk of lung cancer by 1.24 (95%CI 1.18-1.31) in men and 1.12 (95%CI 0.95-1.31) in women. This risk is further increased in smokers.^9^

In 1997, Argentina prioritized asbestos within its National Plan for the Rational Management of Chemical Substances, recognizing that exposure to asbestos poses risks to both workers and the general population. Argentina pledged to provide its citizens with the same level of protection as many developed countries.^10^ In 2000, Argentina became the first country in Latin America to ban the use of asbestos (Resolution 845), specifically amphiboles. In 2001, Resolution 823 banned the production, import, marketing, and use of chrysotile asbestos fibers and products containing them as of 1 January 2003.^11^

Despite these regulatory efforts, the risk of occupational exposure to asbestos persists, particularly for workers involved in demolition, maintenance, repair, and transportation of asbestos-containing structures.^12^ In March 2018, a surveillance program was initiated to monitor the presence of asbestos in the underground facilities of the city of Buenos Aires, Argentina.

The aim of this study was to describe epidemiological monitoring protocols for asbestos and assess the prevalence of occupational diseases related to asbestos exposure among underground workers in Buenos Aires, Argentina.

## METHODS

### DESIGN

We conducted a descriptive cohort study with all underground employees in the city of Buenos Aires. Participants were followed from March 2018 to March 2023.

The study was approved by the Ethics Committee of the British Hospital of Buenos Aires (CRIHB # 1237).

### POPULATION

Underground workers were recruited and categorized as drivers, workshop workers, or other employees. Participants with a follow-up period of less than 36 months were excluded from the analysis.

### PROCEDURE

A surveillance program was implemented to monitor the potential presence of asbestos in facilities, overseen by a multidisciplinary committee. Workers were registered in the Risk Agents Register (RAR), which included employees who had been exposed to asbestos in their workplace. This registration initiated lifelong epidemiological monitoring consisting of 3 specific tests: detection of asbestos detection, chest X-rays, and spirometry, with high-resolution computed tomography (CT) performed as needed. Additionally, clinical records were required to be maintained for 40 years.^13^

**Figure 1:**
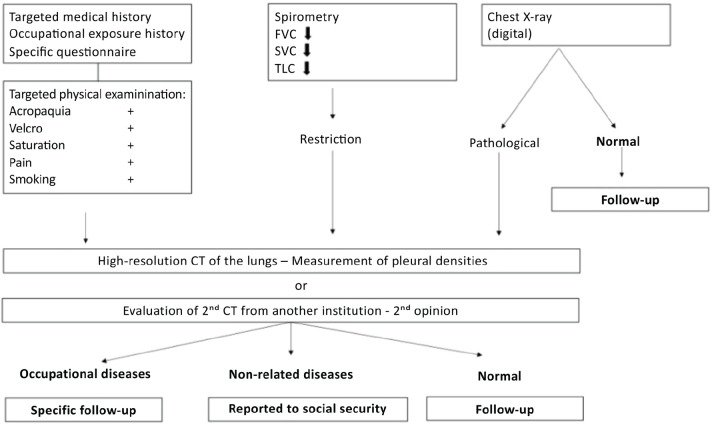
Algorithm for the management, diagnosis, and epidemiological surveillance of workers exposed to asbestos. CT = computed tomography; FVC = forced vital capacity; SVC = slow vital capacity; TLC = total lung capacity, measured by plethysmography.

All participants in this study completed a patient information form and an occupational exposure questionnaire. A specialized team comprising pulmonologists, radiologists, and occupational medicine experts was assembled, and a working algorithm for diagnosis and surveillance was established, as illustrated in Figure 1.

The most significant radiographic indicator considered for registration was the presence of small lung opacities classified as grade 1/1 or higher according to the International Labour Organization (ILO). Additionally, an occupational exposure questionnaire was incorporated into the screening process. A pneumonologist conducted physical examinations to identify signs such as acropachy, Velcro crackles, or other symptoms indicative of asbestos exposure. Spirometry was performed, and if results suggested restriction, this was further confirmed or ruled out through lung volume measurements using plethysmography.

For patients with ambiguous X-ray findings or with clinical and functional abnormalities, high-resolution CT scans with 1-mm thin sections for both inspiratory and expiratory sequences, coupled with the Modified Medical Research Council (mMRC) dyspnea questionnaire, was recommended.

Upon completion of these evaluations, a multidisciplinary team consisting of medical specialists from the union, the occupational risk insurance company, the employer, and the Pneumonology Service of the British Hospital reviewed the findings. Criteria for the indication of chest CT without contrast combining both expiratory and inspiratory sequences was based on the following criteria: a) pathological X-ray findings; b) spirometry suggesting restrictions, confirmed by lung volume measurements; c) presence of Velcro crackles or acropachy; d) unexplained dyspnea; and e) occupational history indicating significant asbestos exposure, as assessed by a specific questionnaire that also evaluated smoking habits.

For patients who completed the study, CT scans were reviewed by a specialized radiologist within the multidisciplinary team. In cases where pleural thickening was observed on the images, the densities were carefully examined to differentiate them from neighboring structures such as extrapleural fat or muscle.

### HISTOPATHOLOGICAL ANALYSIS OF TISSUE BIOPSIES

For patients diagnosed with lung cancer, histopathological analysis was performed on new sections prepared from paraffin-embedded tissue blocks to investigate the presence of asbestos fibers. Hematoxylin and eosin, Giemsa, and Perls stains were used to detect iron deposits and identify asbestos fibers.

### STATISTICAL ANALYSIS

Descriptive statistics were used, with continuous variables expressed as mean ± SD and categorical variables as percentages. Patients were grouped according to the presence or absence of occupational disease. The *t*-test and chi-square test were used to compare variables. Multivariate analysis was performed to determine the impact of exposure duration in years on the onset of asbestos-related disease, with smoking status included as a confounding adjustment variable. Analyses were performed using GraphPad Prism 8.0.2 and MedCalc 12.0.

## RESULTS

**Figure 2:**
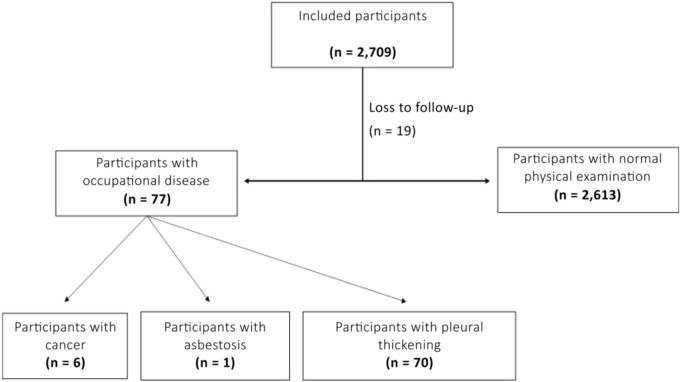
Flow chart of study participants.

A total of 2,709 underground workers in Buenos Aires were initially included in the study (Figure 2). Nineteen participants were excluded due to loss to follow-up. Of the remaining 2,690, 2.8% (n = 77) were diagnosed with an occupational disease, including six cases of cancer (0.22%). The study population was predominantly male (90.1%, n = 2,440), with women accounting for 9.9% (n = 250). Mean participant age was 48.1 ± 10.9 years.

The characteristics of the patients, categorized by the presence or absence of occupational disease, are detailed in Table 1. A significantly higher proportion of occupational disease was observed among workers employed for over 20 years. However, smoking habits did not differ significantly between those with and without occupational disease. Additionally, most symptomatic workers had been diagnosed with an occupational disease, as well as those with restrictive spirometry, pathological X-ray findings, and abnormal CT scans compared to those without occupational disease. Of the 77 patients diagnosed with asbestos-related disease, 94.8% (n = 73) were men, with the remaining 4 being women.

**Table 1:** Characteristics of participants grouped by the presence or absence of occupational disease

	Occupational disease	Normal	p-value
n	77	2,613	
IMC (kg/m^2^)	29.1 ± 7.8	29.4 ± 5.7	0.9
Age	58.1 ± 8.6	24.9 ± 10.9	0.001
Working under 10 years, % (n)	12.9 (10)	28.7 (750)	0.9
Working between 10 to 20 years, % (n)	23.4 (18)	35.3 (923)	0.006
Working over 20 years, % (n)	63.6 (49)	35.9 (940)	0.001
Smoker, % (n)	35.0 (27)	23.5 (614)	0.4
Ex-smoker, % (n)	29.8 (23)	22.4 (585)	0.1
Restrictive spirometry, % (n)	29.8 (23)	7.6 (200)	0.0001
Symptoms, % (n)	74.0 (57)	13.7 (360)	0.001
Pathological CT findings, % (n)	29.8 (23)	6.2 (157)	0.0001
Pathological CX-ray findings, % (n)	29.8 (23)	0.1 (3)	0.0001
Digital clubbing, % (n)	24.7 (19)	1.2 (32)	0.0001
Velcro crackles, % (n)	4.0 (3)	1.2 (32)	0.07
Pain, % (n)	2.5 (2)	0.6 (17)	0.1

BMI = body mass index; CT = computed tomography; CX = chest X-ray.

Six male patients developed malignant neoplasms. Four of them had been exposed to asbestos for more than 20 years, and one for less than 10 years. Among these patients, five were either current or former smokers (Table 2).

**Table 2:** Findings of patients with lung cancer

Age	Smoking (pack/year)	Exposure time (years)	Workplace exposure	Symptoms	Diagnosis
58	40	15	Technical Support Center	Digital clubbing. Chest X-ray: pathological findings.	Adenocarcinoma
55	Never smoked	7	Technical Support Center	Chest pain	Malignant epithelioid mesothelioma
50	25	28	Technical Support Center	No symptoms. Pathological chest X-ray findings.	Adenocarcinoma
51	20	26	Driver	Digital clubbing. Normal spirometry, history of HIV and anal cancer.	Primary lung adenocarcinoma
59	14	25	Technical Support	Pathological findings on chest CT	Acinar adenocarcinoma
50	36	25	Technical Support Center	Digital clubbing and pathological chest X-rays findings	Squamous cell carcinoma

CT = computed tomography.

**Figure 3:**
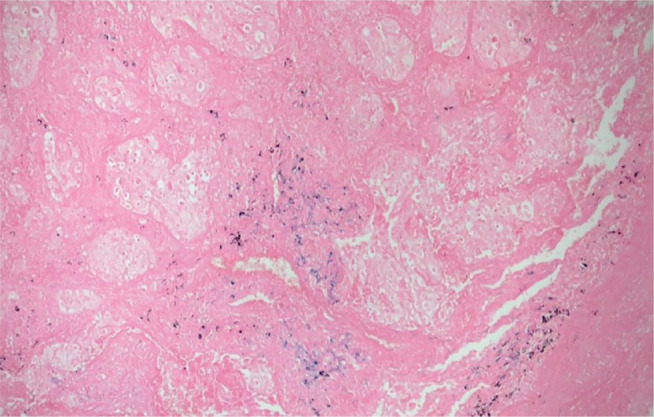
Pathology study. a) Intra-alveolar malignant neoplasm. H&E, original magnification: 10×. b) Alveolar spaces with pneumonocytes, intra-alveolar macrophages, and areas of interstitial hyalinization with anthracotic pigment. H&E: original magnification: 10×. c) Alveolar spaces with pneumonocytes, intra-alveolar macrophages, and areas of interstitial hyalinization with anthracotic pigment. H&E, original magnification: 20×. d) Malignant neoplasm with lymphomononuclear infiltrate and vascular structure thickening.

Light microscopy revealed alveoli containing numerous foamy and multinucleated macrophages, with mesothelioma as the primary diagnosis (Figure 3a). Another patient exhibited areas with ciliated columnar epithelium, sparse macrophages, and malignant neoplasia, diagnosed as squamous cell carcinoma (Figure 3b). In a patient with lung adenocarcinoma, areas of malignant neoplasia coexisted with alveoli showing siderosis and anthracosis (Figure 3c). Lastly, histological sections from another patient with lung adenocarcinoma exhibited fibrohyaline tissue infiltrated with neoplastic cells (Figure 3d).

**Figure 4:**
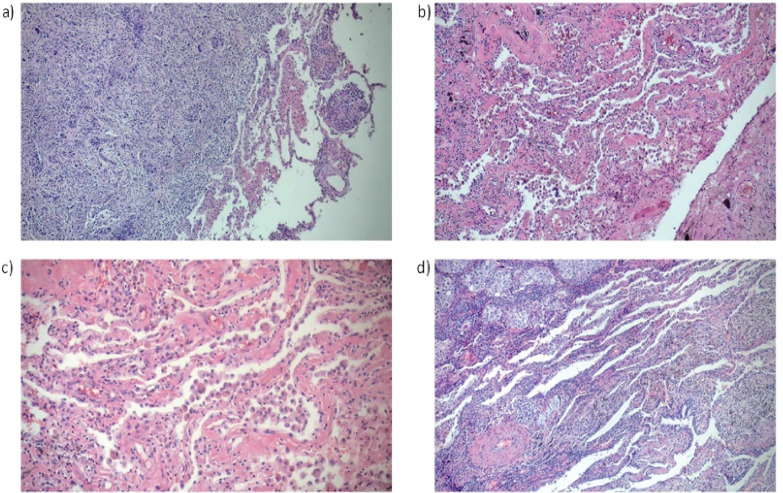
Presence of iron deposits (blue) and anthracotic pigment. Perls’ technique, original magnification: 20×.

Perls’ technique for iron staining did not reveal iron bodies in the “drumstick” form described in the literature. However, iron deposits and anthracotic pigments were detected (Figure 4).

Multivariate analysis determined that occupational exposure for more than 20 years significantly increased the risk ratio for asbestos-related disease (odds ratio [OR]: 3.02, 95%CI 1.72-5.31), while the risk for over 10 years of exposure was OR: 1.28, with 95%CI 0.5832-2.83 (Table 3).

**Table 3:** Multivariate analysis assessing the risk of occupational disease

Variable	Odds ratio	95%CI
Working > 20 years	3.02	1.72-5.31
Working > 10 years	1.28	0.58-2.83
Age	1.04	1.02-1.05

## DISCUSSION

According to the World Health Organization (WHO), approximately 125 million people worldwide are exposed to asbestos in the workplace.^2^ Asbestos consumption in Latin America accounts for 10% of the world’s annual production.^14^ Despite international bans, a global study in 59 countries showed that asbestos-associated mesothelioma causes 9.9 deaths per million annually.^15^

A surveillance program conducted in Germany from 2008 to 2018, involving 2,439 male workers exposed to asbestos, reported a 4.45% prevalence of recognized occupational diseases.^16^ In comparison, the prevalence in our patient cohort was 2.8%. However, the German authors identified a 2.7% prevalence of lung cancer, compared to only 0.22% in our study. Previous research has shown that in Argentina, the risk of mesothelioma due to asbestos exposure is 1.48.^17,18^

The risk of lung cancer increases with asbestos exposure, and the combined impact of asbestos and smoking is cumulative.^19,20^ In our cohort, five of the six patients with lung cancer were smokers. We observed a high percentage of active or former smokers among those with and without occupational disease in our patient series. However, due to the retrospective nature of our study, there may have been potential bias in documenting smoking history for all participants, which precluded its inclusion in the multivariate analysis.

In the multivariate analysis, increased age was also associated with a slight increase in the risk of having an occupational disease. We hypothesized that this trend may be influenced by the number of years of occupational exposure.

Our findings emphasize the need for regular, structured follow-up for all workers with direct or indirect exposure to asbestos. Furthermore, there is a clear need for smoking cessation counseling and a multidisciplinary approach to managing individuals exposed to asbestos in the workplace.
